# Activities and Organophosphate Exposures: Response

**Published:** 2004-09

**Authors:** Gloria D. Coronado, Beti Thompson, Larkin Strong, William C. Griffith, Ilda Islas

**Affiliations:** Cancer Prevention Research Program, Fred Hutchinson Cancer Research Center, Seattle, Washington, E-mail: gcoronad@fhcrc.org

In their letter, Krieger and Zhang note that our article (Coronado et al. 2004) is “founded on an erroneous premise” and that it “presents virtually no data to estimate levels of worker or child exposure.” Our analyses show that the children of workers who reported thinning plants had significantly higher proportions of detectable urinary metabolites of organophosphate (OP) pesticides than the children of workers who reported that they did not perform this task. We concur that no urinary metabolite dose estimates were presented. However, we cited Curl et al. (2002), whose analyses using the same data set show significant correlations between pesticide exposure levels (geometric means and percentiles) between adult farm-workers and children who live in the same household, and high correlations between pesticide residues in house and vehicle dust. When our research team embarked upon investigating the relationship between job task and pesticide exposure, we agreed that calculating the percent detection of metabolites in the urine samples of workers who did and did not perform a given task would permit exploration of this issue. Thus, we noted that the analyses were exploratory in nature (see “Methods”; Coronado et al. 2004). This meant that significant findings would offer directions for further inquiry and investigation.

Krieger and Zhang also state that there is “substantial … data available related to work tasks of handlers,” and they specifically cite the Pesticide Handlers Exposure Database and the U.S. Environmental Protection Agency (EPA) Transfer Coefficients. The Pesticide Handlers Exposure Database aims to determine how much residue (as a percentage of pesticide applied) ends up as external exposure to workers (Van Hemmen 1992). These data are used in worker risk analysis, taking an activity, translating it to external exposure, then translating it to a body burden or toxicologic risk (e.g., using a dermal absorption rates). The database generates hypothesized toxicologic risk estimates and relies on no biomonitoring data, such as testing for pesticide residues in urine or blood. Transfer coefficients estimate the amount of treated foliage with which a worker comes in contact while performing a given task (Knaak et al. 1996). A formula based on fixed assumptions about the clothing that a worker wears and the rate of dermal exposure is used to calculate the body burden of pesticide exposure when a worker performs a given task on a given crop.

We agree that these databases represent important sources of data on both exposure and body burden. However, because formulas used in the calculations of exposure rely on fixed values for protection incurred by personal protective equipment, work hours, absorption rates, and spray patterns, they generate hypothesized risk estimates. Various studies have reported that < 100% of workers routinely use personal protective equipment while applying pesticides, and many may enter recently treated fields (before the expiration of the reentry interval), resulting in potentially higher exposures than estimated in these databases.

Our data are unique in that they provide real-world data on differences in proportion of detection of dimethyl urinary metabolites of adult workers. Moreover, by documenting differences in proportions of detection of urinary metabolites among children of farm-workers, our analyses begin to answer the question of whether or not pesticide residues are being brought into homes where children may be exposed. Because it is widely believed that pesticide residues accumulate in home environments, that they degrade more slowly than pesticides in fields, and that children have unique susceptibilities to and frequencies of exposure (given their propensity for hand-to-mouth behaviors and their frequent contact with floors), such a question merits investigation. Moreover, the relationship between workers’ job task and pesticide residues in collected house dust and vehicle dust samples provide compelling evidence that the take-home pathway is an important source of exposure.

Krieger and Zhang argue that urinary OP metabolite levels of children are more likely linked to dietary exposure than to environmental sources. The findings of Curl et al. (2002)—showing a significant correlation between adult and child urinary metabolite levels and showing lower median total dimethyl urinary metabolite concentrations among children adhering to an organic diet compared to children consuming conventional diets—support the claim that dietary sources contribute to children’s pesticide exposure (Curl et al. 2003). However, there is a growing body of evidence that supports the claim that environmental sources contribute to children’s pesticide exposure. Data from Curl et al. (2002) might also support the take-home pathway because it argues that children are affected by the residues brought home by their parents. McCauley et al. (2001) showed that home pesticide residues in dust are associated with home practices such as changing out of work clothing within 2 hr of returning home. Further research by McCauley et al. (2001)—showing that greater numbers of agricultural workers who live in a house and in close proximity to treated fields is associated with elevated residues of pesticides in house dust—offers additional support for the nondietary pathway of exposure. Our analyses (Coronado et al. 2004) show that the proportion of detectable pesticides residues in home and vehicle dust are greater for workers who thin plants than for workers who do not perform this task (home dust *p-*value = 0.003; vehicle dust *p-*value = 0.001). Although in our analyses we did not assess the associations between dust levels and urinary metabolite concentrations, our results do suggest that contamination of the home environment varies by occupational characteristics (Coronado et al. 2004).

In the next 5 years we will explore the take-home pathways as well as other pathways of pesticide exposure among children, including the dietary pathway. It is our hope that continued research will help clarify the important pathways involved in children’s exposure to pesticides. We believe that the most meaningful and relevant scientific discoveries result from hypotheses that are controversial. We look forward to further research and discovery on this topic.
